# Influence of Intermittent Fasting on Body Composition, Physical Performance, and the Orexinergic System in Postmenopausal Women: A Pilot Study

**DOI:** 10.3390/nu17071121

**Published:** 2025-03-24

**Authors:** Anna A. Valenzano, Paride Vasco, Gabriella D’Orsi, Raffaella R. R. Marzovillo, Maria Torquato, Giovanni Messina, Rita Polito, Giuseppe Cibelli

**Affiliations:** 1 Department of Clinical and Experimental Medicine, University of Foggia, 71100 Foggia, Italy; anna.valenzano@unifg.it (A.A.V.); gabriella_dorsi.579495@unifg.it (G.D.); raffaella.marzovillo@unifg.it (R.R.R.M.); 2 Department of Humanities, University of Foggia, 71100 Foggia, Italy; paride.vasco@unifg.it; 3 Sport Medicine Unit, Policlinico of Foggia, 71100 Foggia, Italy; maria.torquato@unifg.it; 4 Department of Experimental Medicine, University of Campania “Luigi Vanvitelli”, 81055 Santa Maria Capua Vetere, Italy; giovanni.messina@unicampania.it; 5 Department of Psychology and Wellness Sciences, Università Telematica Pegaso, Centro Direzionale Isola F2, 80143 Napoli, Italy; rita.polito@unipegaso.it

**Keywords:** nutrition, diet, exercise, orexin, health promotion

## Abstract

**Objective**: This study aims to evaluate the effects of different nutritional strategies, specifically intermittent fasting (IF) combined with high-intensity interval training (HIIT) versus a low-calorie diet (LCD), on body composition, physical performance, and the orexinergic system in postmenopausal women. **Methods**: A randomized controlled trial involving thirty postmenopausal women (mean age 57.50 ± 6.50 years) was conducted over eight weeks, comparing the two dietary approaches alongside an 8-week HIIT program. Body composition was assessed using bioelectrical impedance analysis (BIA) and dual-energy X-ray absorptiometry (DEXA). Performance metrics included handgrip strength and the 6-min walking test (6MWT). Salivary samples were analyzed for Orexin-A (OX-A) levels pre- and post-intervention. **Results**: Significant improvements in health metrics, such as heart rate (HR) and endurance, were found, with mean HR changes showing a significant difference (F = 5.943, *p* = 0.033) between the groups at T1. Orexin-A levels reflected significant metabolic regulation shifts in relation to other variables, showing a change from baseline to post-intervention values at T1 (F = 10,931, *p* = 0.033). Flexibility (sit and reach) significantly improved by 6% (*p* < 0.05), as well as ${\mathrm{VO}}_{2}$ max (10%, *p* < 0.05), both highlighted as key predictors of overall health outcomes. Additionally, Cohen’s d analyses indicated that the dietary groups exhibited notable differences in endurance, with the LCD group showing a Cohen’s d of −0.90, suggesting a large effect size compared with the control group. **Conclusions**: The combination of IF and HIIT is an effective nutritional strategy for enhancing body composition and physical performance in postmenopausal women, potentially mediated by changes in the orexinergic system. Further research is warranted to explore long-term effects and underlying mechanisms.

## 1. Introduction

Postmenopausal women experience significant physiological changes, including increased visceral fat and decreased muscle mass, primarily due to hormonal shifts, particularly the decline in estrogen. These changes promote systemic inflammation, insulin resistance, and a higher risk of metabolic syndrome, negatively impacting physical function, cardiovascular health, and overall quality of life [1]. Addressing these metabolic disturbances is essential for improving health outcomes in this population, and targeted strategies combining nutrition and exercise have gained increasing attention.

Intermittent fasting has emerged as a promising approach for optimizing body composition and metabolic health. This strategy alternates periods of eating and fasting with popular methods such as the 16:8 protocol (sixteen hours of fasting and an 8-h eating window) and alternate-day fasting [2,3]. Research shows that IF enhances metabolic flexibility and the body’s ability to switch between glucose and fat as primary fuel sources by promoting fat oxidation, improving insulin sensitivity, and reducing inflammation [4,5,6]. These effects are driven by cellular responses to energy deficits, including activating AMP-activated protein kinase (AMPK) and autophagy, improving glucose regulation, mitochondrial function, and oxidative stress resistance [7].

Beyond these metabolic benefits, IF profoundly affects the neuroendocrine system, particularly the orexinergic system. Orexin-A (hypocretin-1), a neuropeptide produced in the lateral hypothalamus, plays a key role in regulating energy balance, arousal, and feeding behavior by activating its receptors, OX1R and OX2R [8]. When energy availability decreases, such as during fasting, orexin neurons respond by stimulating neuropeptide Y (NPY) and agouti-related peptide (AgRP), increasing appetite to restore energy balance while simultaneously activating the sympathetic nervous system (SNS) to boost thermogenesis and lipolysis [9,10,11]. This dynamic coordination helps the body balance energy intake and expenditure in response to metabolic stress.

Importantly, AMPK, a master regulator of cellular energy, activates orexin neurons during fasting, driving lipid metabolism and glucose uptake while promoting mitochondrial biogenesis via peroxisome proliferator-activated receptor γ coactivator 1-alpha [12]. These processes enhance metabolic flexibility, efficiently helping the body transition between fuel sources. The potential for improving mitochondrial function and fat oxidation is directly relevant for postmenopausal women, as it can counteract the metabolic slowdown and visceral fat accumulation associated with menopause [13].

Pairing IF with HIIT may amplify these neuroendocrine and metabolic responses. High-intensity interval training consists of short bursts of intense exercise followed by brief recovery periods, a format known to enhance insulin sensitivity, fat oxidation, and cardiorespiratory fitness [14]. Recent studies suggest that HIIT activates orexinergic pathways by inducing metabolic stress, increasing AMPK activation, and stimulating lipid metabolism and muscle glucose uptake [15,16].

Crucially, fasting and HIIT work synergistically by engaging overlapping metabolic and neuroendocrine pathways. Intermittent fasting promotes metabolic switching, shifting from glucose to fat as the primary fuel, while HIIT triggers acute stress responses via the hypothalamic–pituitary–adrenal (HPA) axis, stimulating cortisol release and orexin activity to support energy mobilization [17]. This dual activation may enhance OX-A levels more effectively than either intervention alone, promoting better energy homeostasis and greater flexibility in fuel utilization. These physiological changes directly translate to practical outcomes for postmenopausal women. Enhanced orexinergic activity supports fat metabolism and muscle function and influences motivation for physical activity by activating the mesolimbic dopamine system, the brain’s reward pathway [18,19]. This link between orexin and motivation suggests that combining IF with HIIT could help improve metabolic health and exercise adherence, an often-overlooked factor in long-term weight management.

Despite these promising connections, the interaction between IF, HIIT, and the orexinergic system remains underexplored, especially in postmenopausal women, whose neuroendocrine activity may already be altered by aging [20]. Given that OX-A plays a pivotal role in regulating energy balance, stress responses, and physical performance, understanding how these interventions modulate orexin activity could open new avenues for tailored strategies to combat metabolic dysfunction and visceral fat accumulation in this population [13].

This study investigates the combined effects of IF and LCD with HIIT on body composition, physical performance, and OX-A levels in postmenopausal women. By exploring the neuroendocrine mechanisms underlying these interventions, the objective is to identify how dietary and exercise strategies influence orexinergic activity and improve metabolic health. This research could inform personalized approaches to enhancing energy regulation, reducing visceral fat, and supporting physical resilience in aging populations.

## 2. Materials and Methods

### 2.1. Study Design

**Figure 1:**
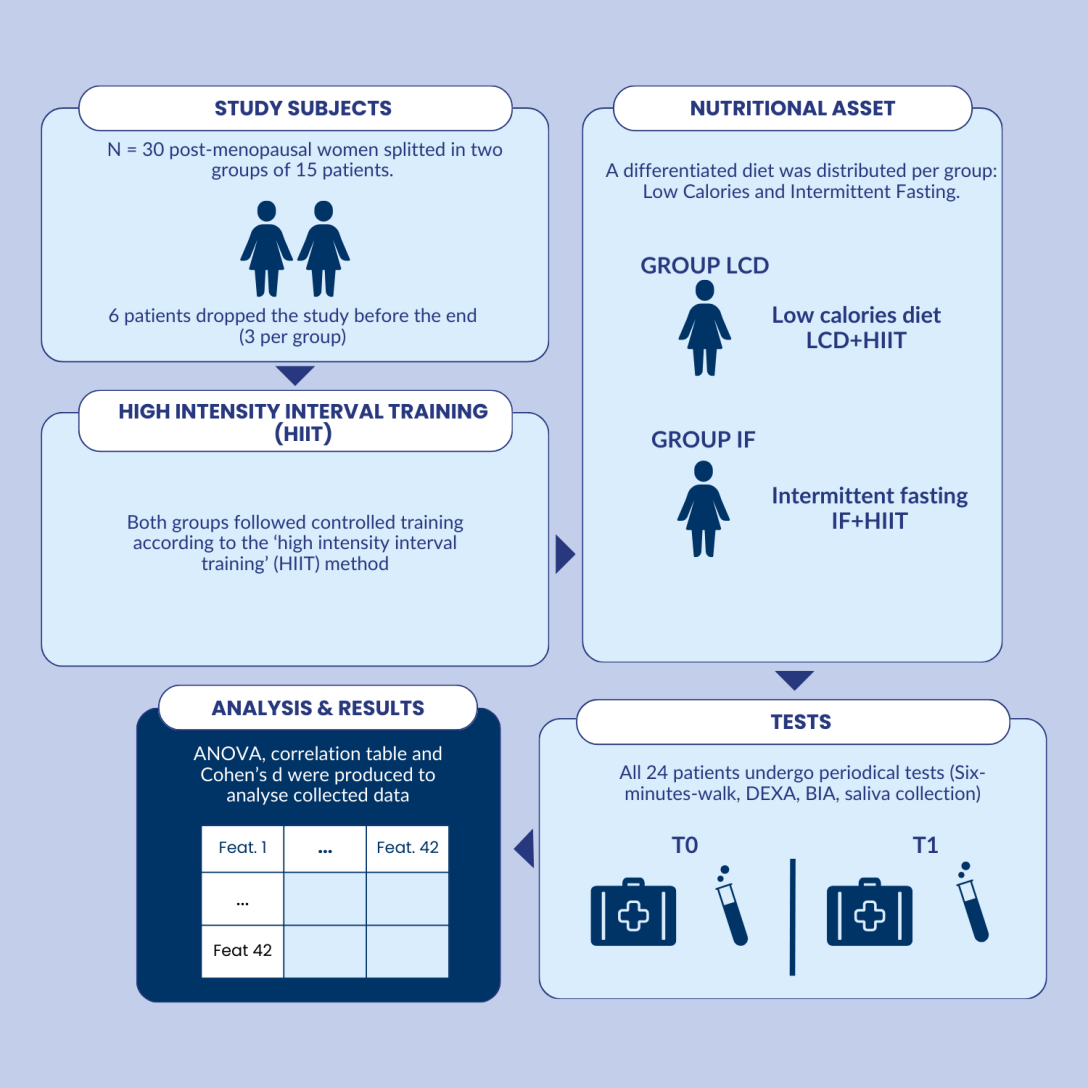
Graphical abstract of our study.

As shown in Figure 1, the study involved thirty participants, equally distributed in two groups, according to their diet preferences. One group followed an IF diet, while the other followed a conventional LCD. Both groups underwent an identical 8-week HIIT training program. The study required all participants to be in good health. Subjects underwent assessment at the baseline (T0) and at the end of the 8-week training period (T1). The study’s outcome measures included body composition assessments, performance evaluations, and analysis of the orexinergic system.

All participants were informed in accordance with the Declaration of Helsinki, and the research was approved by the Ethics Committee of the Policlinico di Foggia (prot, n°440/15—EBTCDI). All participants were fully informed about the study’s purpose, procedures, and potential risks before providing written informed consent. Participation was voluntary, and participants retained the right to withdraw from the study at any time without consequence. Participants had a mean age of 57.5 years (±6.5), a mean height of 160 cm (±3.4), and a mean weight of 69.32 kg (±5.70). To be eligible for inclusion, participants were required to be free from muscular, ligament, bone, nerve, or joint pathologies that could affect the training program, as well as any current cardiovascular or cardiorespiratory issues. Additionally, individuals who were undergoing pharmacological treatment, taking supplements (including caffeine), or participating in other sports activities during the study were excluded from participation.

### 2.2. Body-Composition Assessment

Body composition was assessed using BIA and DEXA, following standardized protocols outlined by previous research [21,22]. Participants were instructed to fast for twelve hours prior to the assessment, wear minimal clothing (underwear), and abstain from exercise for twenty-four hours beforehand to ensure accurate measurements. Body mass index (BMI) was calculated by dividing body weight (kg) by the square of height (m^2^). Body weight was measured using a SECA digital scale (model 877, SECA, Hamburg, Germany) with a precision of 0.1 kg. Height was measured using a SECA stadiometer (model 213, SECA, Hamburg, Germany) with an accuracy of 0.1 cm. Waist circumference (WC) was measured at the narrowest point of the torso, between the lower rib and the iliac crest, while participants stood upright. Measurements were taken following a moderate exhale and recorded to the nearest millimeter. BIA was performed using the Quantum V Segmental device (RJL Systems, Clinton Twp, MI, USA). This method provided estimates of resistance (R, Ω) and reactance (Xc, Ω), which were used to calculate phase angle (PA, °)—a key indicator of cellular health and membrane integrity. Whole-body composition, including total fat mass (kg), lean body mass (kg), and bone mineral content (kg), was assessed using DEXA with the GE Lunar iDXA scanner (GE Healthcare, Madison, WI, USA). A trained researcher (A.V.) conducted all scans following the manufacturer’s guidelines for participant positioning and data acquisition. The Encore software (version 14.1, enCore with CoreScan™) automatically calculated visceral adipose tissue (VAT, g). All measurements were taken in triplicate, and the mean value was used for subsequent statistical analyses.

### 2.3. Orexin-A Analysis

Salivary OX-A concentrations were measured before and after the nutritional intervention using a commercially available enzyme-linked immunosorbent assay (ELISA) kit (Human Orexin A ELISA Kit, Elabscience, Hangzhou, China) following the manufacturer’s instructions, as detailed in Moscatelli et al. [23]. Saliva samples were collected thirty minutes after using Salivette cotton swabs (Sarstedt, Nümbrecht, Germany). Participants were instructed to place the cotton swab in their mouth for two minutes to collect saliva, then return the swab to the collection tube. Samples were immediately frozen at −20 °C until analysis. Orexin-A levels were determined by measuring optical density at 450 nm with a microplate reader (Biotek Power Wave XS; Marshall Scientific, Hampton, NH, USA). Each sample was assayed in triplicate, with all triplicates measured in duplicate.

### 2.4. Performance Evaluations

Physical performance was thoroughly assessed via the 6MWT, along with the handgrip strength test and the sit-and-reach test. In this research, the EN-Motion treadmill (Enraf-Nonius B.V., Rotterdam, The Netherlands) is used to perform a 6MWT in a standardized fashion, allowing it to automatically adjust to the speed of the user. The test measures the distance covered in six minutes on a flat, hard surface in accordance with the ATS guidelines [24]. The results of the test were utilized for both evaluation and training advice. To interpret the participant’s results, the outcome of the test was expressed as a percentage based on the individual’s age, gender, height, and weight. Along with the distance walked various other parameters were recorded during the test, including systolic and diastolic blood pressure at rest and peak effort, maximum and average heart rate (HR), percentage of maximum HR, rate-perceived exertion (RPE), and metabolic equivalents (METs). The maximal oxygen uptake (${\mathrm{VO}}_{2}$ max) was estimated based on the speed and 1% incline of the treadmill, along with some user characteristics (age, weight, height, gender), using the manufacturers’ proprietary algorithms. In this study, treadmill estimations are utilized to monitor changes in the estimated ${\mathrm{VO}}_{2}$ max over time and to compare the relative ${\mathrm{VO}}_{2}$ max of different individuals. The handgrip strength test assessed overall muscle strength [25]. The test was conducted using a mechanical hand-held dynamometer (Jamar, Preston, Jackson, Cape Girardeau County, MO, USA) with a precision of 0.1 kg. The test was conducted with the participant standing with their elbow in full extension and exerting maximum pressure for 2–3 s. Each participant performed the test twice for each hand alternatingly, and the best score from the two attempts was recorded. The test results were used to evaluate changes in muscle strength over time and provide training advice. The sit-and-reach test was used to assess hamstring and lower back flexibility. Participants sat on the floor with their legs fully extended and feet flat against a standardized box, reaching forward as far as possible along a measuring line without bending their knees. The distance reached, measured in centimeters, was recorded as the best of three attempts [26].

### 2.5. Nutritional Intervention

The IF + HIIT group followed a time-restricted feeding protocol using the 16:8 method, where participants consumed all their daily calories within an 8-h eating window, specifically between 7:00 a.m. and 3:00 p.m. Outside of this window, they abstained from all caloric intake for the remaining 16 h, although water, unsweetened tea, and black coffee were permitted. No specific caloric restriction was imposed; participants were instructed to eat ad libitum during the eating window, focusing on balanced meals rich in protein, fiber, and healthy fats. In contrast, the LCD + HIIT group adhered to a continuous calorie-restricted diet without time constraints. Daily caloric intake was individualized based on each participant’s estimated total daily energy expenditure (TDEE), calculated using the Mifflin-St Jeor equation [2] to determine resting metabolic rate (RMR), multiplied by an activity factor. Participants were assigned a diet providing approximately 75% of their TDEE to create a sustained energy deficit. Meals and snacks were evenly distributed throughout the day to ensure steady energy intake, and participants were explicitly instructed to avoid fasting periods. Dietary intake was analyzed using Nutritional Therapy software (version 14.0; DS Medica, Milano, Italy). Participants received written information and participated in a nutritional education session prior to the study to ensure adherence to the IF protocol throughout the intervention.

### 2.6. High-Intensity Interval Training Intervention

The HIIT intervention was conducted over an 8-week period, with participants training three times per week on non-consecutive days, ensuring forty-eight hours of rest between sessions. Each session lasted forty minutes and was supervised by a certified fitness instructor (R.M.) to ensure adherence to the protocol and correct execution of exercises. Each session consisted of three sets of ten repetitions. Participants performed thirty seconds of all-out aerobic exercises, such as sprint intervals or bodyweight exercises (e.g., burpees or squat jumps), followed by thirty seconds of passive rest. The intensity of the aerobic intervals was prescribed based on 75–85% of each participant’s HRmax, calculated using the Tanaka et al. equation [27]. Real-time HR data were continuously monitored using Polar H10 HR monitors (Polar Electro Oy, Kempele, Finland), ensuring that participants maintained the targeted HR zones throughout the sessions. To ensure progressive overload, the program evolved as follows: (i) weeks 1–2: three sets of ten repetitions (thirty s work/thirty s rest), aiming for 75% of HRmax; (ii) weeks 3–5: three sets of ten repetitions, increasing target intensity to 80% of HRmax; (iii) weeks 6–8: three sets of ten repetitions, pushing towards 85% of HRmax. Exercise intensity was individually tailored using participants’ maximal interval efforts, defined as the highest HR reached during an initial incremental exercise test performed in the pre-intervention assessment. This test followed the procedures reviewed by Rosenblat et al. [28], allowing us to calibrate intervals according to each participant’s cardiovascular capacity. Attendance and adherence were carefully tracked, with participants required to attend at least 90% of the scheduled sessions. Any missed sessions were rescheduled within the same week to maintain training consistency.

### 2.7. Statistical Analysis

All statistical analyses were performed using IBM SPSS Statistics, version 23 (IBM Corp., Armonk, NY, USA). Data are presented as M ± SD (mean ± standard deviation). Prior to analysis, the normality of all datasets was assessed using the Shapiro–Wilk test. A power analysis was conducted to ensure a statistical power of 0.85 and an alpha level of 0.05 for detecting differences in the variables of interest, determining that a minimum sample size of nine participants per group was required. To compare baseline characteristics between groups, an independent samples *t*-test was used. Changes from pre- to post-intervention (Δ) were calculated as the difference between the final value and the baseline value for each participant. A two-way repeated measures analysis of variance (ANOVA) was conducted to assess the main effects of time (pre- vs. post-intervention), group (HIIT + IF vs. HIIT + LCD), and the time × group interaction. When a significant effect was found, Tukey’s post hoc test was used to perform pairwise comparisons. To evaluate the strength of observed effects, Cohen’s d was calculated and interpreted as follows: small effect: d < 0.2; moderate effect: d < 0.5; large effect: d < 0.8 [29]. In addition, Pearson’s product-moment correlation coefficients (r) were computed to explore relationships between variables within each group. The significance level for all tests was set at *p* < 0.05.

## 3. Results

The primary aim of this study was to examine the combined effects of IF and LCD, integrated with a HIIT intervention, on body composition, physical performance, and OX-A levels in postmenopausal women. Analysis of the collected data (see Table 1) indicated that at T0, the group following the LCD diet covered a greater average distance (553.33 m) compared with the IF group (480.17 m). However, by T1, the IF group demonstrated a significant increase in performance, achieving an average distance of 610.08 m, which surpassed the 603.50 m covered by the LCD group. This suggests a positive response from the IF group to the combined diet and exercise intervention. In addition, OX-A levels exhibited an intriguing trend. The IF group started with a higher level (53.26 pg/mL) compared with the LCD group (35.18 pg/mL), and these values soared at T1, reaching 143.89 pg/mL for the IF group, while the LCD group increased to only 63.68 pg/mL. This indicates significant metabolic regulation in response to dietary and exercise changes. Furthermore, maximal oxygen uptake and average HR also experienced notable improvements. Specifically, ${\mathrm{VO}}_{2}$ max increased more in the IF group, highlighting the effectiveness of combining HIIT with IF in enhancing aerobic capacity.

An ANOVA (see Table 2) indicated statistically significant differences between groups at T0 and T1.

At T0, significant variations were observed in the 6MWT distance (F = 4.818, *p* = 0.039), mean HR (F = 5.943, *p* = 0.023), and sit-and-reach test (F = 15.849, *p* = 0.001), suggesting baseline differences in flexibility, cardiovascular response, endurance, perceived exertion, and metabolic regulation. At T1, OX-A levels (F = 10.931, *p* = 0.003) remained significantly different between groups, indicating continued differences in perceived exertion and metabolic regulation. Significant changes were also observed in 6MWT distance (F = 4.998, *p* = 0.036), ${\mathrm{VO}}_{2}$ max (F = 5.03, *p* = 0.035), and resistance (F = 6.73, *p* = 0.017), reflecting alterations in body composition, functional exercise capacity, and aerobic capacity.

**Figure 2:**
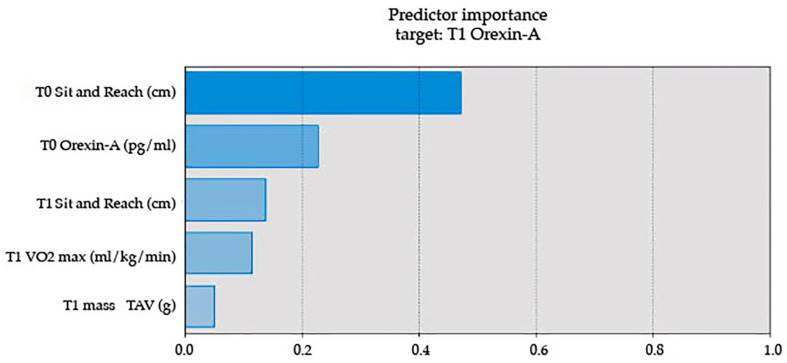
Statistically significant differences among groups at baseline and post-intervention. A coefficient plot in a fan layout shows the significance of each predictor, with bar thickness reflecting weight and color indicating the direction of the association.

**Figure 3:**
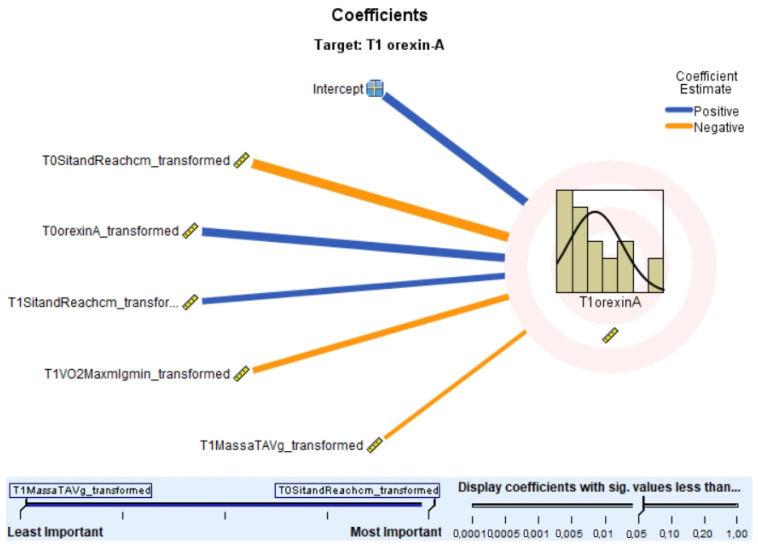
Changes in Predictor Influence on Orexin-A from baseline (T0) to post-intervention (T1). A coefficient plot in a fan layout shows the significance of each predictor, with bar thickness reflecting weight and color indicating the direction of the association. Blue bars indicate a positive impact, while orange bars show a negative effect on post-intervention OX-A.

Key predictors of post-intervention OX-A levels were identified (see Figure 2 and Figure 3). At T0, sit-and-reach test scores and baseline OX-A levels were the strongest positive predictors. At T1, ${\mathrm{VO}}_{2}$ max was a significant negative predictor, suggesting an inverse relationship between aerobic capacity and OX-A levels. The ranking of predictor importance was sit-and-reach test (T0), OX-A (T0), sit-and-reach test (T1), ${\mathrm{VO}}_{2}$ max (T1), and TAV (T1).

**Figure 4:**
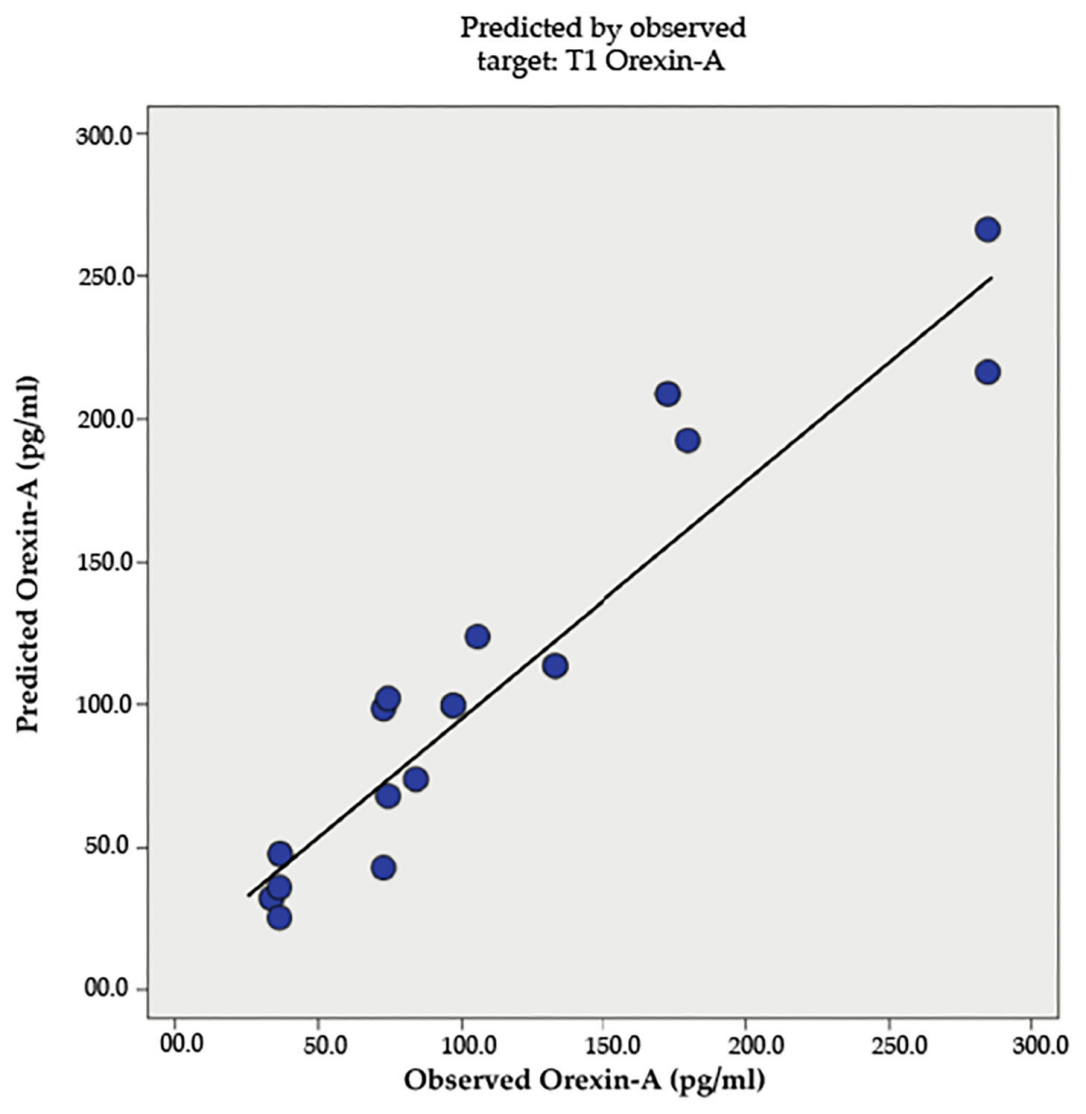
Relationship between observed and predicted Orexin-A levels post-intervention.

A scatterplot (see Figure 4) showed a positive correlation between observed and predicted post-intervention OX-A levels, with some deviations, indicating that the regression model explained part of the variance. Regression analysis confirmed the significance of these predictors, with the sit-and-reach test and baseline OX-A levels positively predicting post-intervention OX-A, and ${\mathrm{VO}}_{2}$ max negatively predicting it.

Regression analysis further identified the key predictors influencing post-intervention OX-A levels. At T0, the sit-and-reach cm transformed emerged as the most significant positive predictor, closely followed by the baseline OX-A transformed. In contrast, at T1, ${\mathrm{VO}}_{2}$ max mL/g/min transformed negatively impacted OX-A levels, indicating a possible inverse relationship between aerobic capacity and orexinergic activity post-intervention. Table 3 provides a detailed analysis of the key predictors of post-intervention OX-A levels, highlighting the interconnected effects of flexibility, metabolic function, and aerobic fitness. Pearson correlation analysis (see Table 3) revealed significant associations between physiological and performance measures. T0 mean HR was negatively correlated with T1 OX-A levels (r = −0.573, *p* = 0.033), and T0 6MWT distance was positively correlated with the T0 mean HR (r = 0.611, *p* = 0.033).

Cohen’s d analyses (see Table 4) showed distinct effect sizes across dietary groups IF and LCD at T0 and T1. At T0, the LCD group showed superior endurance capacity (distance covered, d = −0.90) and flexibility (sit and reach, d = −1.63), while the IF group showed higher aerobic capacity (${\mathrm{VO}}_{2}$ max, d = 0.36), perceived exertion tolerance (RPE, d = 0.77), and OX-A levels (d = 0.73). At T1, the IF group showed greater improvements in RPE (d = 1.72), ${\mathrm{VO}}_{2}$ max (d = 0.92), and sit-and-reach (d = 0.32), while the LCD group showed better adaptations in reactance (d = −1.46). The most significant shift was in sit-and-reach performance for the IF group (Δd = 1.94). Orexin-A levels showed a moderate positive shift for the IF group (Δd = 0.62).

These findings offer critical insights into how nutritional strategies and exercise interventions influence physical performance and metabolic biomarkers in postmenopausal women, emphasizing the role of flexibility and initial OX-A levels as key predictors in understanding these adaptations. At T0, Cohen’s d values indicated differing advantages between the IF and LCD groups across various metrics. A negative effect size for distance covered (d = −0.90) favored the LCD, suggesting superior baseline endurance capacity in this group. Conversely, ${\mathrm{VO}}_{2}$ max (d = 0.36) and RPE (d = 0.77) showed a performance advantage for the IF group, indicating higher initial aerobic capacity and perceived exertion tolerance. The sit-and-reach test exhibited a substantial adverse effect size (d = −1.63), implying greater baseline flexibility in the LCD group. In contrast, OX-A levels showed a moderate positive effect size (d = 0.73), favoring the IF group, suggesting a potential metabolic advantage in orexinergic activity at the study’s onset. Following the intervention, Cohen’s d analyses revealed significant shifts, particularly favoring the IF group in key physiological measures. Rate-perceived exertion (d = 1.72) and ${\mathrm{VO}}_{2}$ max (d = 0.92) demonstrated substantial improvements in the IF group, reflecting enhanced aerobic fitness and exercise tolerance. Although the sit-and-reach test effect size shifted positively (d = 0.32), the change was minor, indicating a less pronounced improvement in flexibility over time. The most striking shift in Cohen’s d values was observed in post-intervention sit-and-reach performance (Δd = 1.94), highlighting a significant improvement in flexibility for the IF group. Conversely, reactance values (Δd = −1.46) shifted toward the LCD, suggesting better adaptations in bioimpedance measures for this group. Additionally, OX-A levels exhibited a moderate positive shift (Δd = 0.62), reinforcing the increasing metabolic effect of the IF diet over time.

## 4. Discussion

This study examined the physiological and neuroendocrine effects of IF and LCD in postmenopausal women, highlighting their impact on body composition, physical performance, and OX-A dynamics. By integrating a HIIT program, this study explores how dietary strategies and exercise interventions modulate metabolic and hormonal responses, offering more profound insights into the orexinergic system’s role in energy balance, cardiovascular function, and physical performance.

Our results show that both IF and LCD produced distinct metabolic benefits, but the addition of HIIT appeared to amplify neuroendocrine adaptations. A significant increase in post-intervention OX-A levels was observed, particularly in the IF + HIIT group. Since OX-A is a key neuropeptide regulating energy homeostasis, arousal, and feeding behavior, this rise suggests a direct link between intense physical activity, fasting states, and enhanced orexinergic activity. The observed increase in OX-A likely reflects the combined effects of intermittent metabolic switching, induced by IF, and acute stress responses (triggered by HIIT), a finding consistent with Messina et al. [11], who emphasized orexin’s role in promoting fat oxidation and insulin sensitivity. Orexin neurons in the hypothalamus are highly sensitive to energy deficits, becoming more active during fasting and exercise to stimulate lipid metabolism and glucose uptake via the AMPK pathway [11]. This aligns with existing research indicating that IF activates the “metabolic switch,” shifting the body from glucose to lipid metabolism, which enhances fat oxidation and improves metabolic efficiency [30].

Furthermore, IF has been shown to reduce oxidative stress and inflammation, likely mediated by metabolic switching and the upregulation of cellular repair mechanisms [1]. The fat mass reduction observed in the IF group mirrors findings by Varady et al. [4], who reported significant fat loss following alternate-day fasting.

The LCD group also demonstrated substantial reductions in fat mass and improvements in metabolic markers, consistent with Stockman et al.’s research [6]. The LCD’s effect appears tied to caloric restriction and the metabolic adaptation of using ketone bodies as an alternative energy source, potentially contributing to the anti-inflammatory responses noted in previous studies [31]. However, LCD may have a differential long-term impact compared with IF. The sustained caloric deficit inherent in LCD can lower energy expenditure over time, a known metabolic adaptation that may explain the weight-loss plateau some participants experienced, aligning with observations by Harris et al. [7].

Our study also shed light on the orexinergic system’s broader role in metabolic regulation. Beyond its impact on fat metabolism and glucose uptake, orexin modulates the HPA axis, a crucial stress response pathway. The activation of orexin neurons during HIIT likely stimulated cortisol release, which can drive lipid mobilization and energy expenditure [18]. This dual activation of orexin and HPA pathways suggests that the IF + HIIT protocol triggers a hormonal stress response: repeated, low-level stressors, like fasting and intense exercise, provoke adaptive neuroendocrine changes that enhance metabolic flexibility and resilience [32].

A novel finding of this study was the link between baseline sit-and-reach performance and post-intervention OX-A levels. Our regression analysis showed that greater flexibility positively predicted OX-A responses, a relationship that may stem from the orexin system’s role in muscle glucose uptake and energy regulation. This association echoes research by Kim et al. [33], suggesting that musculoskeletal health can influence metabolic outcomes. These findings broaden the view of physical fitness, indicating that beyond strength and endurance, flexibility may also shape hormonal responses and metabolic efficiency.

Interestingly, ${\mathrm{VO}}_{2}$ max at the end-point negatively predicted OX-A levels, pointing to a complex interaction between aerobic capacity and orexinergic activity. While HIIT improved cardiorespiratory fitness, the inverse relationship with OX-A may reflect a neuroendocrine balancing act: as aerobic capacity rises, the body may rely less on orexin signaling, shifting towards other metabolic regulators like catecholamines and glucocorticoids [20]. This nuanced relationship highlights the need for further research into how fitness markers interact with neuroendocrine pathways.

Our correlation analysis revealed significant links between cardiovascular fitness, physical performance, and OX-A levels. The negative correlation between baseline HR and post-intervention OX-A suggests that participants with lower initial cardiovascular fitness experienced greater orexinergic activation after IF and HIIT. This may indicate a compensatory response, where individuals with lower fitness rely more heavily on orexin neurons to balance energy demands during fasting and intense exercise. The positive correlation between distance covered and baseline HR further supports the idea that HR dynamics mirror neuroendocrine activity, especially when HIIT challenges the body to regulate energy expenditure and stress responses.

Cohen’s d analysis offered valuable insights into the practical significance of these neuroendocrine changes. At baseline, the IF group exhibited higher ${\mathrm{VO}}_{2}$ max and perceived exertion, suggesting a greater reliance on orexin-driven energy pathways. Post-intervention, the IF + HIIT group showed dramatic improvements in RPE and ${\mathrm{VO}}_{2}$ max, reinforcing the idea that fasting and high-intensity exercise bolster aerobic capacity and neuroendocrine resilience.

The most striking change was seen in sit-and-reach performance, supporting the emerging link between flexibility and metabolic health, a relationship potentially mediated by the orexinergic system’s regulation of muscle glucose uptake [34].

These findings are particularly relevant for postmenopausal women, a population often facing hormonal imbalances, visceral fat accumulation, and reduced neuroendocrine activity. The combination of IF and HIIT offers a dual-action strategy, enhancing metabolic flexibility while stimulating the orexinergic system to mitigate the metabolic decline associated with menopause [33]. By improving insulin sensitivity, reducing inflammation, and boosting cardiovascular health, these strategies may help manage obesity-related conditions prevalent in this demographic. Ultimately, this study highlights the neuroendocrine benefits of pairing IF with HIIT, demonstrating how these interventions work together to regulate energy homeostasis, metabolic function, and physical performance through orexin-mediated pathways.

As OX-A continues to emerge as a crucial player in metabolic and hormonal health, future research should investigate how diet and exercise strategies can further optimize neuroendocrine responses, unlocking new ways to promote metabolic health and longevity in aging populations.

While this study highlights the physiological and neuroendocrine benefits of combining IF, LCD, and HIIT in postmenopausal women, several limitations should be considered. First, the short intervention period may not fully capture the long-term neuroendocrine adaptations or the sustainability of these dietary and exercise strategies. While notable increases in OX-A levels suggested heightened energy regulation and metabolic flexibility, it remains unclear whether these effects persist over extended periods or if the body adapts to prolonged fasting and high-intensity exercise in ways that may alter orexinergic activity. Future studies should adopt longitudinal designs to explore how these interventions impact hormonal and metabolic pathways over time.

Second, although this study focused exclusively on postmenopausal women, gender differences in neuroendocrine responses to both diet and exercise warrant further investigation. Research shows that hormonal fluctuations, such as those involving estrogen and cortisol, can modulate orexin signaling and energy homeostasis [20]. Understanding these differences is crucial, as men and women may exhibit varying metabolic and neurohormonal adaptations to HIIT and fasting protocols.

Moreover, the healthy cohort used in this study may limit the generalizability of our findings to metabolically challenged populations, such as individuals with type 2 diabetes, obesity, or metabolic syndrome, who may experience dysregulated orexin pathways. Given the orexinergic system’s role in insulin sensitivity, fat oxidation, and energy balance, future research should investigate how these interventions affect those with impaired metabolic function.

Another limitation lies in the psychological factors influencing dietary adherence and exercise performance. While OX-A is known to modulate reward pathways and cravings, it did not directly assess how hunger, satiety, or mood fluctuations impacted participant behavior. Since HIIT and fasting may trigger both physiological stress responses via the HPA axis and psychological stress due to caloric restriction, future studies should integrate behavioral measures to better understand the neuropsychological dimensions of these interventions.

Finally, although the synergistic effects of HIIT and IF are acknowledged, which appear to amplify orexinergic activity and enhance metabolic regulation, it did not empirically isolate the effects of exercise alone versus diet alone. This leaves a gap in understanding how each component contributes independently and interactively to neuroendocrine outcomes. Future research should employ multifactorial designs to dissect the specific and combined roles of HIIT, fasting, and caloric restriction, tailoring interventions to individuals’ metabolic profiles and fitness levels. Ultimately, addressing these limitations will pave the way for personalized strategies that harness the neuroendocrine benefits of diet-exercise combinations, offering targeted solutions for improving metabolic health, energy regulation, and physical performance in both clinical and healthy populations.

## 5. Conclusions

This study highlights the interconnected metabolic, physiological, and neuroendocrine benefits of IF and LCD in postmenopausal women, emphasizing their effects on body composition, physical performance, and OX-A regulation. Both dietary strategies contributed to fat mass reduction and improved metabolic health, with IF enhancing metabolic flexibility and fat oxidation and LCD supporting fat loss through sustained caloric restriction. Crucially, both interventions positively influenced OX-A levels, linking dietary strategies to energy regulation and metabolic efficiency. The combination of IF and HIIT emerged as a particularly effective approach, showing a synergistic effect on visceral fat reduction, aerobic capacity, and orexinergic activity. This suggests that pairing targeted dietary strategies with structured exercise programs not only optimizes fat metabolism but also activates neuroendocrine pathways, offering a powerful strategy for improving physical performance and metabolic resilience in postmenopausal women. Furthermore, Cohen’s d analysis reinforced the practical significance of these interventions, highlighting meaningful improvements in key physiological markers. These findings present a compelling and sustainable approach to the management of obesity-related conditions and metabolic dysfunction during menopause, offering targeted strategies to counteract the hormonal and metabolic changes that often accompany this stage of life. Looking ahead, future research should focus on long-term neuroendocrine adaptations to these interventions, exploring how orexin pathways interact with individual metabolic profiles and how psychological factors, such as appetite regulation and exercise motivation, influence adherence and outcomes. Unraveling these complex relationships will be crucial in developing personalized diet-exercise programs that support metabolic health and functional performance in aging populations.

## Figures and Tables

**Table 1:** Comparison between the two dietary groups (IF and LCD) for key measured parameters at two time points (T0 and T1).

Measure	IF Mean	IF St. Dev.	LCD Mean	LCD St. Dev.
T0 Distance (m)	480.17	96.68	553.33	63.14
T1 Distance (m)	610.08	36.13	603.50	33.81
T0 Evaluation (%)	79.67	18.78	83.50	15.11
T1 Evaluation (%)	101.08	10.10	90.58	12.75
T0 ${\mathrm{VO}}_{2}$ max (mL/kg/min)	27.07	2.85	25.70	4.56
T1 ${\mathrm{VO}}_{2}$ Max (mL/kg/min)	32.77	3.51	29.87	2.78
T0 HR mean (bpm)	109.33	21.12	127.67	15.25
T1 HR mean (bpm)	117.25	15.62	121.25	10.50
T0 Orexin-A (pg/mL)	53.26	30.73	35.18	16.75
T1 Orexin-A (pg/mL)	143.89	81.47	63.68	20.63
T0 Sit and Reach (cm)	8.42	3.15	14.42	4.17
T1 Sit and Reach (cm)	16.92	6.33	15.25	3.96
T0 Weight (kg)	66.29	4.28	63.92	9.37
T1 Weight (kg)	64.08	5.97	63.54	9.05

**Table 2:** Significant ANOVA results for the various measured variables. Our elaboration.

Test Group	Sum of Squares	df	Mean Square	F	*p*-Value
**T0 Distance (m)**					
Between Groups	32,120.17	1	32,120.17	4.82	0.033
Within Groups	146,674.33	22	6667.01		
Total	178,794.50	23			
**T0 HR mean (bpm)**					
Between Groups	2016.67	1	2016.67	5.94	0.023
Within Groups	7465.33	22	339.33		
Total	3482.00	23			
**T0 Sit and Reach (cm)**					
Between Groups	216.00	1	216	15.85	0.001
Within Groups	299.83	22	13.63		
Total	515.83	23			
**T1 Evaluation (%)**					
Between Groups	661.50	1	661.50	4.99	0.036
Within Groups	2311.83	22	132.85		
Total	313.02	23			
**T1 ${\mathrm{VO}}_{2}$ Max (mL/kg/min)**					
Between Groups	50.46	1	50.46	5.03	0.035
Within Groups	220.81	22	10.04		
Total	271.27	23			
**T1 Orexin-A (pg/mL)**					
Between Groups	38,607.48	1	38,607.48	10.93	0.003
Within Groups	77,700.51	22	3531.84		
Total	116,307.99	23			
**T1 Resistance (Ω)**					
Between Groups	10,045.04	1	10,045.04	6.73	0.017
Within Groups	32,846.92	22	1493.04		
Total	1633.96	23			

**Table 3:** Results of Pearson correlations for various test pairs with two-tailed significance.

Test Pair	Statistic	Value
T0 Distance (m)—T0 HR mean (bpm)	Pearson Correlation	0.611
	*p*-value (2-tailed)	0.002
T0 Distance (m)—T0 Sit and Reach (cm)	Pearson Correlation	0.426
	*p*-value (2-tailed)	0.038
T0 Distance (m)—T1 Orexin-A (pg/mL)	Pearson Correlation	−0.667
	*p*-value (2-tailed)	0.000
T0 HR Mean (bpm)—T0 Sit and Reach (cm)	Pearson Correlation	0.612
	*p*-value (2-tailed)	0.001
T0 HR Mean (bpm)—T1 Orexin-A (pg/mL)	Pearson Correlation	−0.573
	*p*-value (2-tailed)	0.003
T0 Sit and Reach (cm)—T1 Orexin-A (pg/mL)	Pearson Correlation	−0.492
	*p*-value (2-tailed)	0.015
T1 Evaluation (%)—T1 ${\mathrm{VO}}_{2}$ Max (mL/kg/min)	Pearson Correlation	0.668
	*p*-value (2-tailed)	0.000
T1 Evaluation (%)—T1 RPE	Pearson Correlation	0.422
	*p*-value (2-tailed)	0.400
T1 Evaluation (%)—T1 Resistance (Ω)	Pearson Correlation	−0.805
	*p*-value (2-tailed)	0.000
T1 RPE—T1 Resistance (Ω)	Pearson Correlation	−0.511
	*p*-value (2-tailed)	0.011

**Table 4:** Cohen’s d values for T0, T1 measurements and the change ΔT for IF Group and LCD Group.

Variables	T0	T1	ΔT
Distance (m)	−0.90	0.19	1.09
Evaluation (%)	−0.22	0.91	1.13
${\mathrm{VO}}_{2}$ max (mL/kg/min)	0.36	0.92	0.56
RPE	0.77	1.72	0.95
Sit and Reach (cm)	−1.63	0.32	1.95
Orexin-A (pg/mL)	0.73	1.35	0.62

## Data Availability

The original contributions presented in the study are included in the article and Supplementary Materials, further inquiries can be directed to the corresponding author.

## Supplementary Materials

The following supporting information can be downloaded at: https://www.mdpi.com/article/10.3390/nu17071121/s1.
