# Supplementary figures and images for: Influence of Intermittent Fasting on Body Composition, Physical Performance, and the Orexinergic System in Postmenopausal Women: A Pilot Study
Source: Nutrients. 2025 Mar 24;17(7):1121. doi: 10.3390/nu17071121 (PMC11990565; doi:10.3390/nu17071121)

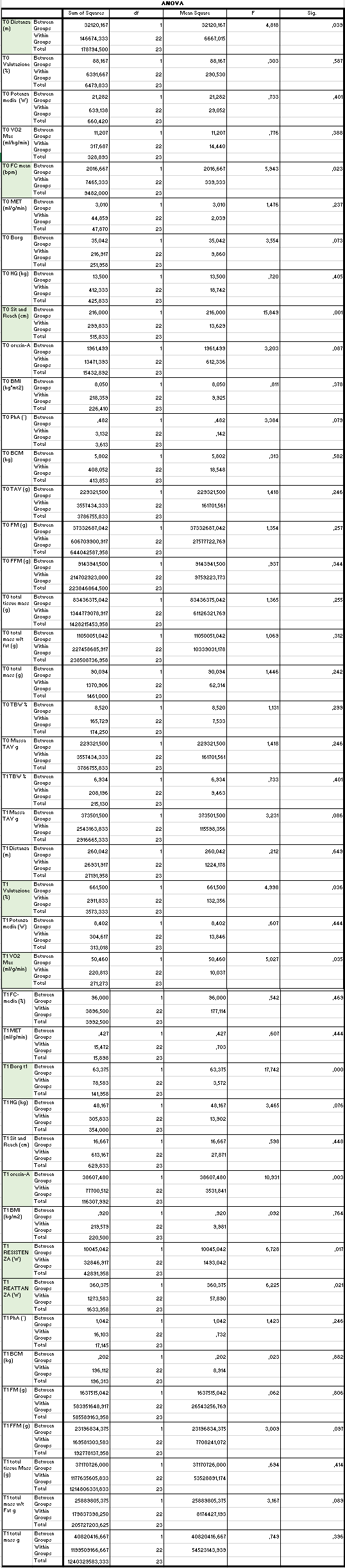

Supplement: Supplementary file 1 [file nutrients-17-01121-s001.zip › Figure S1.png]

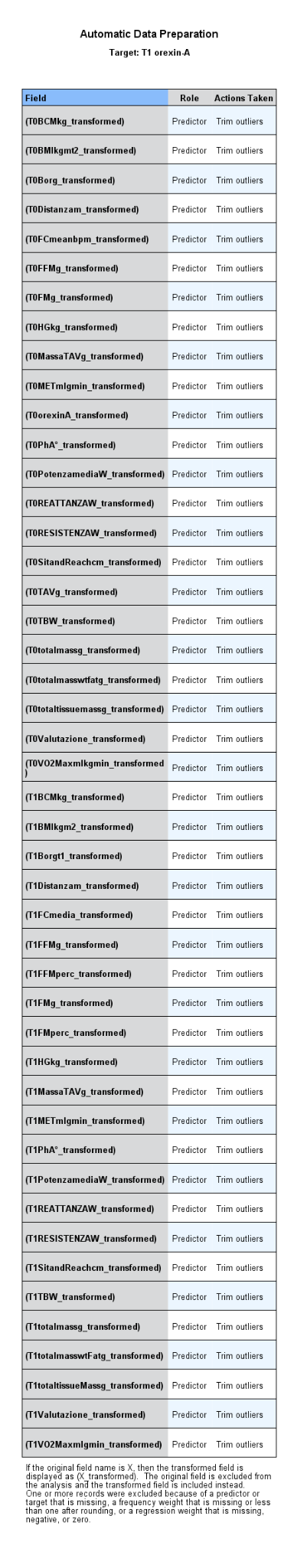

Supplement: Supplementary file 1 [file nutrients-17-01121-s001.zip › Figure S2.png]

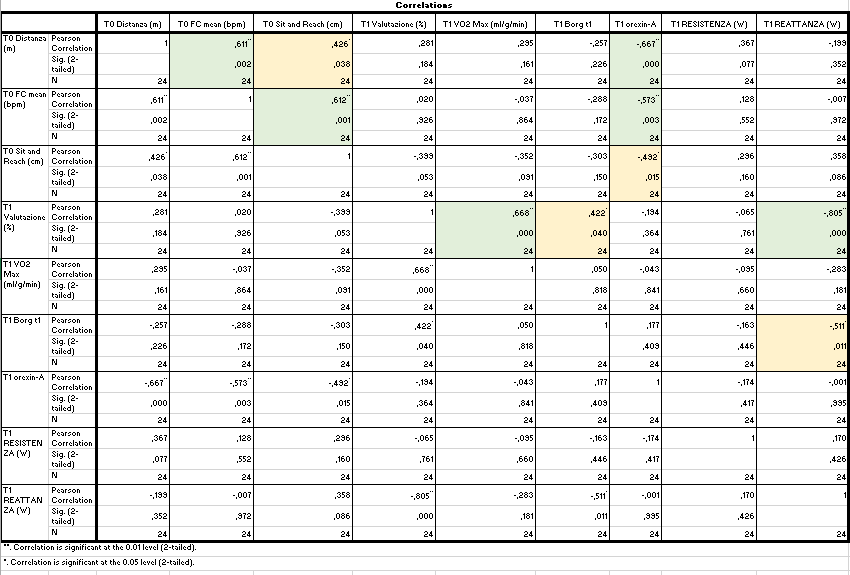

Supplement: Supplementary file 1 [file nutrients-17-01121-s001.zip › Figure S3.png]

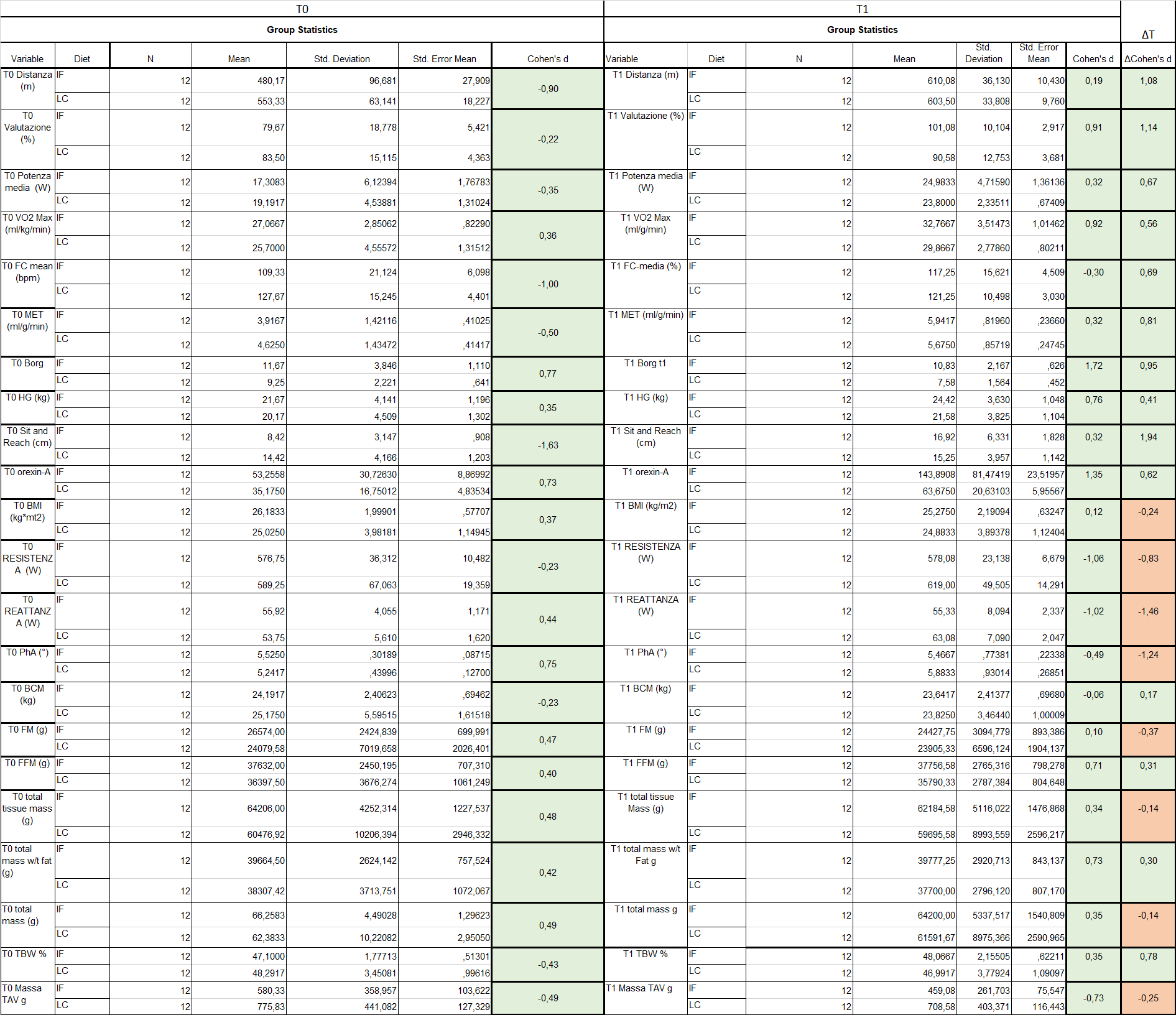

Supplement: Supplementary file 1 [file nutrients-17-01121-s001.zip › Figure S4.png]
